# Functional Analysis of Human Hematopoietic Stem Cell Gene Expression Using Zebrafish

**DOI:** 10.1371/journal.pbio.0030254

**Published:** 2005-07-05

**Authors:** Craig E Eckfeldt, Eric M Mendenhall, Catherine M Flynn, Tzu-Fei Wang, Michael A Pickart, Suzanne M Grindle, Stephen C Ekker, Catherine M Verfaillie

**Affiliations:** **1** Department of Medicine, Division of Hematology, Oncology, and Transplantation, and Stem Cell Institute, University of Minnesota, Minneapolis, Minnesota, United States of America,; **2** Genetics, Cell Biology, and Development and Arnold and Mabel Beckman Center for Transposon Research, University of Minnesota, Minneapolis, Minnesota, United States of America,; **3** Cancer Center Bioinformatics Division, University of Minnesota, Minneapolis, Minnesota, United States of America; Harvard Medical School and Children's HospitalUnited States of America

## Abstract

Although several reports have characterized the hematopoietic stem cell (HSC) transcriptome, the roles of HSC-specific genes in hematopoiesis remain elusive. To identify candidate regulators of HSC fate decisions, we compared the transcriptome of human umbilical cord blood and bone marrow CD34^+^CD33^−^CD38^−^Rho^lo^c-kit^+^ cells, enriched for hematopoietic stem/progenitor cells with CD34^+^CD33^−^CD38^−^Rho^hi^ cells, enriched in committed progenitors. We identified 277 differentially expressed transcripts conserved in these ontogenically distinct cell sources. We next performed a morpholino antisense oligonucleotide (MO)-based functional screen in zebrafish to determine the hematopoietic function of 61 genes that had no previously known function in HSC biology and for which a likely zebrafish ortholog could be identified. MO knock down of 14/61 (23%) of the differentially expressed transcripts resulted in hematopoietic defects in developing zebrafish embryos, as demonstrated by altered levels of circulating blood cells at 30 and 48 h postfertilization and subsequently confirmed by quantitative RT-PCR for erythroid-specific *hbae1* and myeloid-specific *lcp1* transcripts. Recapitulating the knockdown phenotype using a second MO of independent sequence, absence of the phenotype using a mismatched MO sequence, and rescue of the phenotype by cDNA-based overexpression of the targeted transcript for zebrafish *spry4* confirmed the specificity of MO targeting in this system. Further characterization of the *spry4*-deficient zebrafish embryos demonstrated that hematopoietic defects were not due to more widespread defects in the mesodermal development, and therefore represented primary defects in HSC specification, proliferation, and/or differentiation. Overall, this high-throughput screen for the functional validation of differentially expressed genes using a zebrafish model of hematopoiesis represents a major step toward obtaining meaningful information from global gene profiling of HSCs.

## Abstract 2

Microarray studies are combined with a functional screen in zebrafish to identify new genes that are involved in the function and differentiation of hematopoietic stem cells.

## Introduction

Hematopoiesis is the process by which hematopoietic stem cells (HSCs) give rise to all hematopoietic lineages during the lifetime of an individual. To sustain lifelong hematopoiesis, HSCs must self-renew to maintain or expand the HSC pool [1], and they must differentiate to form committed hematopoietic progenitor cells (HPCs) that progressively lose self-renewal potential and become increasingly restricted in their lineage potential. A combination of extrinsic and intrinsic signals are thought to converge to regulate HSC differentiation *versus* self-renewal decisions, but the molecular mechanisms that regulate these processes are poorly understood [2].

A multitude of cytokines have been cloned that affect HSCs and HPCs; however, to date none of these, alone or in combination, can induce the symmetrical, self-renewing HSC cell division in vitro that is required for HSC expansion. Recently, several novel regulators of HSC fate decisions have been identified. For instance, overexpression of *Hoxb4* results in expansion of murine and human HSCs with an increased competitive repopulation potential [3–5]; novel extrinsic regulators implicated in self-renewal of HSCs include Notch [6], Wnt [7,8], and the morphogens, *SHH* (sonic hedgehog) [9] and *BMP4* (bone morphogenetic protein 4) [9]. While the discovery of these novel regulators provides credence to the hypothesis that extrinsic and intrinsic signals can influence HSC fate, a more global gene and/or protein expression analysis of human HSC should provide additional insight into pathways that support HSC self-renewal.

Our current understanding of the expressed gene profile of HSCs comes primarily from murine HSCs that can be purified to near homogeneity [10–14]. The inability to purify human HSCs to similar degrees of homogeneity makes study of the transcriptome of human HSCs more difficult. Human HSCs and HPCs are CD34 positive, while cells that engraft in severe combined immunodeficiency (SCID) mice are enriched in the CD34^+^Lineage(Lin)^−^CD38^−^ fraction [15]. As fewer than 1/500 CD34^+^Lin^−^CD38^−^ cells can repopulate SCID mice [15], the expressed gene profile of CD34^+^Lin^−^CD38^−^ cells is likely only partially enriched for HSC-specific genes [12,16]. We previously demonstrated that the rhodamine (Rho) 123^−^ and c-kit^+^ subpopulation of CD34^+^Lin^−^CD38^−^ cells (Rho^lo^) cells are highly enriched for primitive HPCs with myeloid–lymphoid initiating cell (ML–IC) capacity relative to CD34^+^CD38^−^CD33^−^Rho^hi^ (Rho^hi^) cells [17]. Thus, such selection separates CD34^+^Lin^−^CD38^−^ cells into HSC-enriched and HSC-depleted populations.

We hypothesized that comparison of the transcriptome of Rho^lo^ and Rho^hi^ cells from umbilical cord blood (UCB) and bone marrow (BM) should identify conserved genes and gene pathways that define the human HSC. Because of the inherent limitations of using gene expression data to infer biological gene function, we also assessed the hematopoietic role of these genes in a high-throughput in vivo functional genomics screen in the zebrafish. Using this strategy we have not only identified a series of genes that may represent novel regulators of human HSC fate decisions, but this work also represents, to our knowledge, the first example of a functional genetic screening strategy that is a critical step toward obtaining biologically relevant functional data from global gene-profiling studies.

## Results/Discussion

### ML–ICs Are Highly Enriched in Rho^lo^ Compared to Rho^hi^ Cells

The study of human HSCs has been limited since the CD34^+^Lin^−^CD38^−^ fraction of hematopoietic cells, commonly used as an HSC-enriched population, contains fewer than 0.2% SCID-repopulating cells [15], suggesting considerable heterogeneity. We have shown that ML–IC s, single hematopoietic cells that can generate several daughter cells capable of reinitiating long-term myeloid and long-term lymphoid cultures, are highly enriched by selecting the Rho^lo^ fraction of CD34^+^Lin^−^CD38^−^ cells. While the Rho^lo^ population still only contains 15%–25% ML–ICs and therefore remains heterogeneous, the enrichment factor is 5- to 10-fold greater than CD34^+^Lin^−^CD38^−^ cells [17]. Similar to our previous studies, the ML–IC frequency was greater than or equal to 10-fold higher in UCB Rho^lo^ compared to Rho^hi^ cells (Figure S1).

### Genes Differentially Expressed between Rho^lo^ and Rho^hi^ Cells from Both UCB and BM

**Figure 1:**
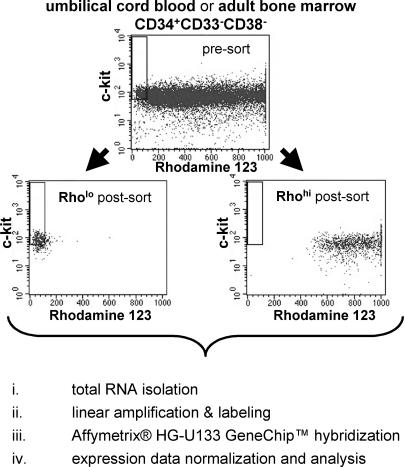
Fluorescence-Activated Cell Sorting and Gene Expression Analysis UCB or adult BM CD34^+^CD33^−^CD38^−^Rho^lo^c-kit^+^ (Rho^lo^; stem cell enriched) and CD34^+^CD33^−^CD38^−^Rho^hi^ (Rho^hi^; stem cell depleted) cell populations were sorted for subsequent global gene expression profiling. Total RNA was isolated from Rho^lo^ and Rho^hi^ cell populations prior to linear amplification and labeling for hybridization to the Affymetrix HG-U133 GeneChip set (approximately 45,000 probe sets) and subsequent data analysis.

We hypothesized that comparing genes differentially expressed between Rho^lo^ and Rho^hi^ cells from ontogenically distinct sources would identify conserved genes and gene pathways that govern self-renewal and differentiation of human HSCs. The experimental design used is illustrated in Figure 1. We defined differentially expressed probe sets as those with *p* < 0.05, using a paired Student's *t*-test. By taking into account the variability present in primary cell populations, this provides a more accurate analysis of differential gene expression compared with the commonly used fold change cutoff.

We identified 2,707 and 4,667 probe sets differentially expressed between Rho^lo^ and Rho^hi^ cells from UCB and BM, respectively (see Tables S1 and S2). The fidelity of our microarray results was confirmed using quantitative RT-PCR (Q-RT-PCR) (see Figure S2). We focused our further analysis on 277 unique transcripts, represented by 304 probe sets that were differentially expressed between Rho^lo^ and Rho^hi^ cells from both UCB and BM with a fold change greater than 1.5 in either UCB or BM (Table S3).

Among the conserved genes enriched in Rho^lo^ cells, many have been implicated in early hematopoiesis, including *CDKN1A*, a cell cycle regulator required for maintenance of murine HSCs [18], and *ABCB1*, the ABC-transporter family member responsible for the Rho^lo^ phenotype [17]. Several transcription factors (TFs) known to play a role in early hematopoiesis or leukemogenesis were also identified, including *HLF*, involved in leukemogenic chromosomal translocations [19] and *EVI1*, a TF associated with myeloid leukemias [20]. Other TFs without a known role in hematopoiesis were also more highly expressed in Rho^lo^ cells, including *HMGA2*, a high-mobility group gene, and the zinc finger TFs *ZNF165*, *ZNF331*, and *KLF5*. All Rho^lo^-enriched genes are listed in Table S3. As demonstrated in previous HSC gene-profiling studies [12–14], more than 40% of genes enriched in Rho^lo^ cells lack a functional annotation, are hypothetical proteins, or are expressed sequence tags, and thus may represent currently uncharacterized regulators of HSC fate decisions (Figure S3).

Some genes with well-established roles in HSC self-renewal and early differentiation are not present in the Rho^lo^ enriched gene list. However, most of these were differentially expressed in both datasets, but differences did not reach statistical significance. For instance, *LMO2* [21] and *GATA* *2* [22], known to be involved in HSC development and self-renewal, were expressed significantly higher in BM Rho^lo^ than in Rho^hi^ cells. Although similar trends were seen in UCB Rho^lo^ cells, these differences were not statistically significant (Table S4). Conversely, *HOXB4* [4] expression was significantly higher in UCB Rho^lo^ than in Rho^hi^ cells, but this difference was not statistically significant in BM. Although our stringent criteria for differential expression likely contribute to the omission of some genes that might be differentially expressed, another explanation might be that expression of these genes is maintained when cells differentiate from a Rho^lo^ to a Rho^hi^ stage. The latter is consistent with the fact that most known HSC-associated genes were expressed at much higher levels than the normalized average microarray expression level in Rho^lo^ and in Rho^hi^ cells from both UCB and BM (Table S4).

Conserved genes enriched in the Rho^hi^ cells included *LEF1*, an effector of Wnt signaling expressed in pre-B and T cells [23], and *NOTCH2*, involved in hematopoietic differentiation cell-fate decision [24]. Several TFs known to play a role in hematopoietic cell differentiation were more highly expressed in Rho^hi^ compared to Rho^lo^ cells, including *HELLS* and *MAFB* [25,26]. Additional TFs with no known role in hematopoietic development were also enriched in Rho^hi^ cells, such as the zinc finger homeobox gene, *ZFHX1B* and the polycomb genes, *EZH2* and *SUZ12*, the latter required for germ cell development [27]. Globin *(Hb)* gene family members were also more highly expressed in UCB and BM Rho^hi^ than in Rho^lo^ cells. Consistent with the ontogenic expression patterns of fetal *versus* adult *Hb* genes, *Hbγ* genes were more highly expressed in perinatal UCB Rho^hi^ cells, while *Hbβ* genes were more highly expressed in adult BM Rho^hi^ cells. Additional genes enriched in Rho^hi^ cells are listed in Table S3.

Because of functional redundancy among gene families, we examined the data for common differentially expressed gene family members. The Id family of transcriptional repressors [28] was enriched in the Rho^lo^ fraction, but was represented by different family members in UCB *(ID4)* and BM *(ID1*, *ID2*, and *ID3).* Similarly, various H1 and H2 histone genes were enriched in the Rho^lo^ fraction in both datasets, but were represented by distinct family members.

We also evaluated whether common differentially expressed genes were concentrated on specific chromosomes. We found that genes were not only concentrated on certain chromosomes, but at specific g-band addresses. Of the genes enriched in Rho^lo^ cells, 9% reside at 6p21, a region involved in recurrent chromosomal translocations in myeloid [29] and lymphoid [30] leukemias, and home to the *PIM1* oncogene [31]. Six members of the H2B and one member of the H1 histone family, as well as *CDKN1A*, more highly expressed in Rho^lo^ than Rho^hi^ cells, reside at 6p21. The remaining Rho^lo^-enriched genes at 6p21 consist of six class II major histocompatibility complex (MHC) family members and a putative testis-specific zinc finger TF, *ZNF165*. H1 and H2 histone gene family members [11,13], class II MHC antigens [12,13], and *CDKN1A* [13] were also found among the genes identified in studies characterizing the transcriptome of murine HSC. The differential expression of such a large number of genes located at this chromosomal address suggests that, like *CDKN1A*, other genes located at 6p21 with as yet unknown hematopoietic function may play a role in HSC proliferation or differentiation.

We also compared the genes expressed more highly in Rho^lo^ *versus* Rho^hi^ cells with published gene expression data. Comparison with the study by Ivanova et al. [12] that compared human CD34^+^Lin^−^CD38^−^ with CD34^+^Lin^−^CD38^+^ cells, yielded only seven genes in common: *ARMCX2*, *CRYGD, HLF*, *KIAA1102*, *RBPMS*, *SLCO3A1*, and *SSBP2*. The lack of overlap may not be that surprising as Rho^lo^ and Rho^hi^ cells are subpopulations of the CD34^+^Lin^−^CD38^−^ population used by Ivanova et al. Comparison of genes expressed more highly in Rho^lo^ *versus* Rho^hi^ cells with genes expressed more highly in murine side population/KLS/CD34^−^ compared to total BM cells published by Ramalho-Santos et al. [13] identified 16 likely orthologs and 38 common gene family members (Table S5), suggesting that HSC-specific genes are conserved across species.

#### In vivo functional genomics screen in zebrafish

**Figure 2:**
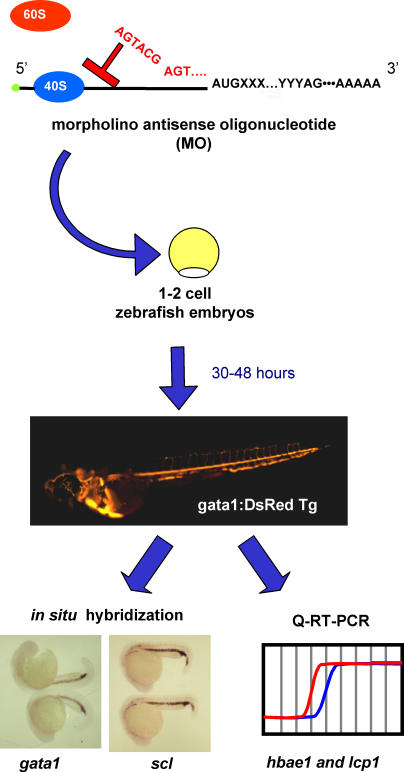
Functional Genomics Screen in Zebrafish The hematopoietic function of differentially expressed candidate genes was determined by injecting MOs into one- to two-cell embryos from *gata1:*DsRed Tg zebrafish to disrupt gene expression. Injected embryos were scored at 30–48 hpf for the presence of DsRed^+^ blood cells by fluorescence microscopy. Subsequently, MO-targeted embryos with gross hematopoietic defects were analyzed for the expression of the early hematopoietic markers *gata1* and *scl* by whole-mount in situ hybridization, and the late hematopoietic markers *hbae1* and *lcp1* by Q-RT-PCR.

Because gene profiling per se does not prove functional importance, we developed an in vivo functional genomics screen in zebrafish (Figure 2). The zebrafish, *Danio rerio*, is an ideal organism for high-throughput genetic screens [32] as organogenesis is highly conserved from zebrafish to man [33]. There is abundant evidence that hematopoiesis in zebrafish occurs via a highly conserved genetic program. As in mammals, hematopoiesis in zebrafish occurs via specification of mesoderm to a hemangioblast stage that subsequently commits to either HSC or angioblasts [34], and genes and signals involved in specification (BMP signaling) and commitment (vascular endothelial growth factor signaling; *flk1*, also known as *kdr; lmo2; scl*, also known as *tal1; gata2;* *gata1*) are conserved from fish to man [35]. This high degree of homology in the genetic control of zebrafish and human hematopoietic development makes genetic screens in zebrafish a powerful tool to elucidate the role of genes in hematopoiesis. Additionally, rapid reverse genetic screens can be accomplished using morpholino antisense oligonucleotides (MOs) to knock down gene expression in the developing zebrafish embryo [36].

**Figure 3:**
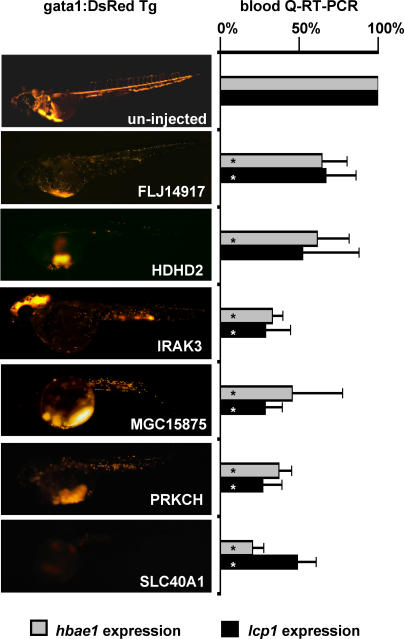
Representative Hematopoietic Phenotypes Observed in MO-Targeted Zebrafish Fluorescence microscopic images of *gata1*:DsRed Tg zebrafish embryos display the hematopoietic phenotypes observed for six MO-targeted zebrafish embryos compared to an uninjected control Phenotypes shown are representative of greater than 70% of injected embryos at 48 hpf (greater than or equal to three experiments of *n* > 40 embryos). Hematopoietic defects were quantified by Q-RT-PCR for the expression of erythroid-specific *hbae1* and myeloid-specific *lcp1* transcripts in MO-targeted embryos relative to uninjected clutchmate controls (greater than or equal to three experiments of *n* = 5 embryos; * *p* < 0.05)

**Figure 4:**
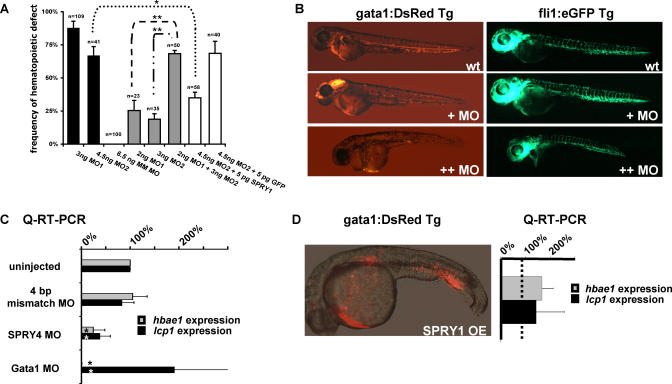
spry4 Is Required for Normal Hematopoietic Development in Zebrafish Embryos (A) The observed frequency of hematopoietic defects in *gata1:*DsRed Tg zebrafish embryos are indicated for two independent *spry4*-targeted MOs (MO1 and MO2; black bars), a four-base mismatched control MO (MM MO; no bar indicates a 0% frequency), a low-dose injection of MO1 and MO2 individually and in combination (gray bars), and MO2 coinjected with a human *SPRY1* DNA expression vector or a GFP control (white bars) (error bars = standard deviation of the mean, *p < 0.05, **p ≤ 0.01). (B) Representative pictures of the phenotypes seen with *spry4* MO1 at 48 h using *fli1:*EGFP/*gata1:*DsRed double Tg zebrafish embryos that have EGFP^+^ vascular endothelial cells and DsRed^+^ erythroid cells. The embryos display a more drastic reduction in DsRed^+^ blood cells with some blood pooling and pericardial edema at higher MO doses (++MO) without major vasculature defects. The *gata1:*DsRed and *fli1:*EGFP Tg images are representative of three experiments of *n* > 40 embryos each. (C) Q-RT-PCR analysis of *hbae1* and *lcp1* transcripts in *spry4* MO1, control mismatch MO and a *gata1* MO-injected zebrafish at 48 hpf (*p < 0.05). (D) Injection of 30 pg of human *SPRY1* DNA expression vector resulted in an expansion of DsRed^+^ hematopoietic cells in the posterior ICM in greater than 50% of successfully injected embryos at 32 hpf (three experiments of *n* = 30), and a representative bright-field image is pictured with the fluorescence micrograph overlaid (left). Q-RT-PCR analysis of *hbae1* and *lcp1* transcripts in *SPRY1* overexpressing embryos relative to uninjected clutchmate controls (three experiments of *n* = 5 embryos) (right).

From the 277 unique transcripts that were differentially expressed between Rho^lo^ and Rho^hi^ cells of both UCB and BM (Table S3), we eliminated genes with known function in hematopoiesis, MHC genes, histones, and genes that are known to play a role in glucose and protein metabolism and RNA and DNA synthesis, resulting in a final list of 158 genes. Of these, we identified a putative zebrafish ortholog for 86, and designed MOs against 61 (Table S6). The 61 MOs were injected in one- to two-cell zebrafish embryos and assayed for effects on blood development. Initial dosing studies identified 16/61 MOs that reduce blood cell production without confounding toxicities (Table 1). The 16 MOs induced a blood defect in more than 70% of embryos in two or more independent injections. Blood defects identified by *gata1:*DsRed transgenic (Tg) fluorescence microscopy were confirmed by Q-RT-PCR of the erythroid-specific *hbae1* and myeloid-specific *lcp1* transcripts in MO-targeted embryos compared to uninjected controls in three or more independent experiments of *n* = 5 embryos per experiment. A greater than 2-fold reduction in both erythroid and myeloid gene expression levels were seen for five of seven MOs analyzed (see Table 1; Figures 3 and 4). Thus, the addition of Q-RT-PCR to the screening process provides an independent confirmation and quantitation of the observed phenotypes, thereby limiting the false-positive rate, while maintaining the high-throughput nature of the screen. Additionally, the observed reduction of both erythroid and myeloid gene expression following knockdown of candidate genes is consistent with their presumed roles in HSC fate decisions prior to specification of the common myeloid progenitor. The validity of the Q-RT-PCR analysis was corroborated by analysis of *hbae1* and *lcp1* transcript levels in *gata1* MO-targeted embryos, in which there was a virtually complete loss of *hbae1* expression and an almost 2-fold increase in myeloid-specific *lcp1* transcripts (Figure 4) consistent with the published expression patterns for these genes following loss of *gata1* expression [37]. Analysis of vascular development by injecting MOs into *fli1:*enhanced green fluorescent protein (EGFP) Tg embryos, which express EGFP in endothelial cells, demonstrated no major abnormalities in vascular morphogenesis or remodeling that precludes the circulation of the remaining blood cells, indicating that the hematopoietic defect is not secondary to a vascular phenotype (Figure 4; Table S7).

**Table 1:**
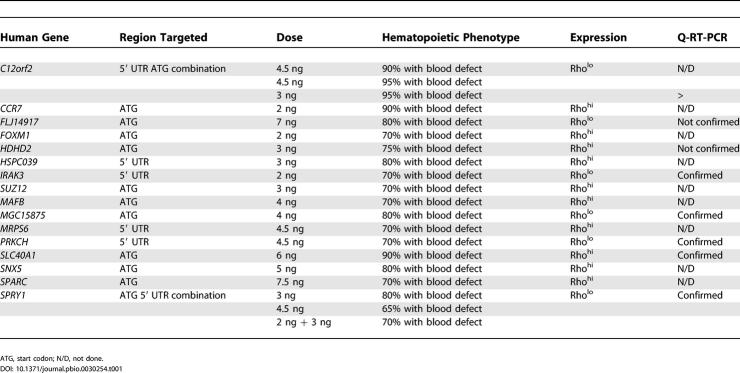
Genes Differentially Expressed between Human Rho^lo^ and Rho^hi^ Cell Populations That Have a Functional Role in Zebrafish Hematopoietic Development

The greater than 20% frequency of blood defects seen in our screen compares very favorably with the 0.5%–1% frequency of hematopoietic phenotypes seen by ethylnitrosourea mutagenesis screens that mutate genes in a near random fashion [38] and the 4% of hematopoietic phenotypes seen in a morpholino-based functional screen of the zebrafish secretome [39] (S. C. Ekker, unpublished data). The high incidence of blood defects also demonstrates that the candidate genes identified by comparing the transcriptome of Rho^lo^ and Rho^hi^ cells represent genes with important roles in HSC biology. We were unable to identify a candidate ortholog in zebrafish for 72/158 of the differentially expressed human genes. This may be because currently only one quarter of the zebrafish genome is high-quality finished sequence. Hence, some genes with important roles in hematopoiesis may have been untested. However, from a sample of ten genes that lacked a zebrafish match, only one has a likely ortholog in the Fugu or Medaka sequencing projects, and thus incomplete genome coverage provides only a partial explanation. Alternative possibilities include a reduced level of primary sequence conservation between functional orthologs that would have been missed using our simple comparative genomics criteria, or that a number of genes are not conserved between fish and man, and thus might be less important for the conserved processes of hematopoietic self-renewal and differentiation. Therefore, the relatively high incidence of blood defects in conserved genes may in part reflect “evolutionary filtering” in our screen. Consistent with this hypothesis, all zebrafish genes whose mutation resulted in a visible embryonic phenotype identified using a retroviral insertion strategy have a likely human ortholog [40].

Although the frequency of blood defects is high, the screen is not as well suited for the identification of knockdown phenotypes that result in increased HSC proliferation and/or differentiation. We and others have successfully shown that dramatically increased hematopoietic development can be modeled in zebrafish, as is the case for knockdown of the BMP-antagonist *chd (chordin)* and the corresponding *dino* mutant [41,42]; however, more modest increases in hematopoietic cell production or skewing of lineage differentiation may be undetectable. Although these caveats may lead to underestimation of the true frequency of genes with a role in early hematopoietic development and differentiation, the screening procedure used has proven effective for extracting functional information from a global gene expression profiling dataset in a high-throughput manner.

Of note, viable knockout mice exist for 4/14 genes identified in our functional screen *(Sparc, Irak3, Ccr7*, and *Prkch)* [43–46]. One could argue that if a viable knockout mouse exists, the gene of interest may not be important in hematopoiesis. However, lack of an overt hematopoietic phenotype does not preclude a role of a gene in HSC self-renewal and differentiation, as this may only be detectable under conditions where the hematopoietic system is stressed or in transplantation experiments. For example, *Hoxb4* ^−/−^ mice develop normally and present with only subtle differences in spleen and BM cellularity [47]. However, the proliferative response of HSC in vitro and in vivo is decreased, consistent with the observation that overexpression of *Hoxb4* supports expansion of competitive repopulating units and SCID-repopulating cells [4,5].

### Characterization of *Sprouty* Family Members in Zebrafish Hematopoiesis

To further verify the hematopoietic role of genes identified by gene array, we performed a more extensive evaluation of the zebrafish targeted with an MO against *spry4* (spry4 morphant or spry4^MO^). Although human *SPRY1* was differentially expressed, an MO against zebrafish *spry4* was used, as it is expressed in the region of the lateral plate mesoderm, the first site of zebrafish hematopoiesis [48], and it was the full-length zebrafish *Sprouty* gene with the greatest protein homology to human *SPRY1*. Recently the partial sequence of a potential zebrafish *spry1* ortholog was predicted by Ensembl's gene prediction software based on genomic sequence information. However, the single exon that was predicted does not contain an ATG start codon, or a conserved splice donor or acceptor site, and the putative zebrafish *spry1* sequence only partially covers the human *SPRY1* gene. Moreover, the genomic location of Ensembl's putative zebrafish *spry1* is currently not known, and therefore it is not possible to use syntenic relationships to determine the most likely zebrafish ortholog for human *SPRY1*. At present, there is not sufficient sequence data available to design gain- or loss-of-function experiments for the putative zebrafish *spry1*, thus precluding an analysis of hematopoietic function in the zebrafish model. Therefore, *spry4* is currently the best full-length, MO-targetable candidate ortholog for human SPRY1, and based on our results, at the very least zebrafish *spry4* and *SPRY1* share a conserved function in hematopoiesis.

To confirm the specificity of MO targeting in the spry4^MO^, a second *spry4* MO of independent sequence and a four-base mismatched *spry4* MO were injected into zebrafish embryos. Injection of the independent *spry4* MOs induced a hematopoietic phenotype in more than 65% of injected embryos, while the four-base mismatched MO did not induce any phenotypic changes (Figure 4). Q-RT-PCR for *hbae1* and *lcp1* transcripts also did not show changes in expression in four-base mismatched MO embryos. Furthermore, the two independent *spry4* MOs acted synergistically when coinjected (Figure 4). In addition to the blood phenotype, a slight facial outgrowth was seen at a low frequency. Also, a weak dorsalization phenotype was seen at 2-fold higher MO doses based on a decreased somite size.

To rule out the possibility that the hematopoietic defect observed in the spry4^MO^ was secondary to a vascular defect, we injected *spry4* MO into *fli1:*EGFP Tg zebrafish. While the resulting embryos exhibited minor defects in cardinal vein remodeling and morphogenesis of intersegmental vessels in the posterior tail, there were no major defects in vascular development (Figure 4). The integrity of the vascular network in spry4^MO^ fish was further demonstrated by the unimpeded circulation of the remaining DsRed^+^ blood cells in *gata1:*DsRed Tg spry4^MO^ (data not shown).

Thisse et al. [35] have shown that overexpression of zebrafish *spry4* mRNA leads to an expansion of the posterior intermediate cell mass (ICM) [48]. We overexpressed human *SPRY1* in *gata1:*DsRed Tg zebrafish embryos, and observed a similar dose-dependent expansion of DsRed^+^ blood cells in the posterior ICM (Figure 4). Q-RT-PCR analysis of embryos overexpressing *SPRY1* revealed a 1.7- and 1.4-fold increase *hbae1* and *lcp1* mRNA levels, respectively, confirming the observed expansion of blood cells in the ICM. Coinjection of human *SPRY1* cDNA with *spry4* MOs ameliorated the spry4^MO^ phenotype, yet another confirmation of the specificity of MO targeting (Figure 4). The similar hematopoietic phenotypes observed following the overexpression of either human *SPRY1* or zebrafish *spry4* indicate that these genes encode proteins with a similar functional potential in blood development. In addition to the blood phenotype, the overexpression often caused a reduction and/or curve in the posterior tail (Figure 4) not seen in the overexpression of zebrafish *spry4* [48].

**Figure 5:**
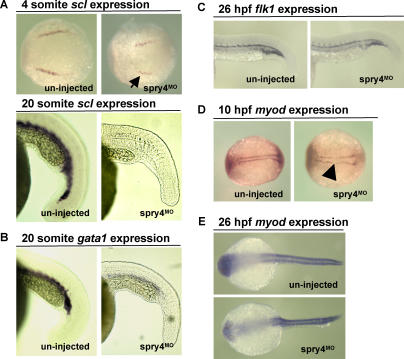
spry4 Is Required for Early Hematopoietic Development, but Not Mesodermal Commitment, in Developing Zebrafish Embryos (A) Expression of the early hematopoietic marker *scl* was reduced at the four-somite stage (arrow) and virtually absent at the 20-somite stage in spry4^MO^ embryos compared to uninjected controls. (B) The more mature hematopoietic marker *gata1* was also reduced in spry4^MO^ compared to uninjected zebrafish embryos at 20 somites. (C and D) In contrast to hematopoietic genes, the mesodermal-specific *flk1* (vasculature) transcripts were expressed at similar levels to uninjected controls at 26 hpf, and *myod* (muscle) had a slight defect (arrowhead) at 10 hpf, while expression was similar to controls at 26 hpf. All images are representative of greater than or equal to two experiments of *n* ≥ 8 embryos.

Characterization of hematopoietic gene expression in the spry4^MO^ by whole-mount in situ hybridization revealed a reduction in *scl* expression at four somites (8/15), and virtually no *scl* (12/20) or *gata1* (18/25) expression at 20 somites (Figure 5), consistent with a defect in mesodermal commitment to HSCs and/or HSC proliferation and differentiation. The few hematopoietic cells that are present in the morphant are hemoglobinized based on *o*-dianisidine staining (data not shown). To determine whether the hematopoietic defects observed in spry4^MO^ were the result of a defect in mesoderm specification during development, we performed whole-mount in situ hybridization for the vasculature-specific *flk1* and muscle-specific *myod* transcripts. At 10 h postfertilization (hpf) there was a slight defect in *myod* expression (5/10), while *myod* expression at 26 hpf was comparable to wild type (21/21) (Figure 5). This suggests that there were no major defects in mesodermal commitment in the spry4 morphants. Considering the nearly absent expression of the early hemangioblast and HSC cell marker *scl*, these results may suggest that the defect induced in the spry4^MO^ occurs prior to HSC specification. However, the normal expression pattern of other mesodermal genes, such as *flk1* and *myod* at 26 hpf in the spry4^MO^ (Figure 5), indicate that the hematopoietic phenotype is not merely a consequence of defective specification of mesoderm. Finally, the hematopoietic gene *cmyb*, a presumed marker of definitive HSC in zebrafish [49], was absent at 38 hpf (10/12) (data not shown), suggesting that the spry4^MO^ embryos are devoid of definitive HSC.

In vertebrates, *Sprouty* family members act as antagonists for fibroblast growth factor (FGF), vascular endothelial growth factor, and epidermal growth factor signaling, and they may be involved in feedback regulation, as *Sprouty* gene expression is induced by activation of these signaling pathways [50]. *Sprouty* genes antagonize receptor tyrosine kinase signaling at the level of the Ras/Raf/mitogen-activated protein kinase pathway; however, they also can serve as positive regulators of these pathways in some cell types [50]. Therefore, our current hypothesis is that *SPRY1* may affect HSC by modulating FGF-mediated, perhaps in combination with other receptor tyrosine kinase-mediated, signaling. In fact, three of the 14 genes that induce a hematopoietic defect in the zebrafish screen, *SPRY1*,*MAFB* and *SPARC*, are all involved in FGF signaling [50–52], thus suggesting a role for FGF in hematopoiesis. Studies are ongoing to confirm a role of *SPRY1* in mammalian hematopoiesis, by testing the effect of overexpression of *SPRY1* on the repopulating ability of human and murine HSCs. Similar definitive studies in zebrafish and subsequent confirmation in mammalian HSC models are underway for genes that were functionally validated in our zebrafish screen. We believe that the sequential genetic screen in zebrafish followed by confirmation in mammalian models as described here will establish a hematopoietic function for genes identified by gene array analysis in a high-throughput and efficient manner.

## Materials and Methods

### Isolation of Rho^lo^ and Rho^hi^ cell populations from UCB and BM

Human UCB from full-term delivered infants and BM from healthy donors were obtained after informed consent in accordance with guidelines approved by the University of Minnesota Committee on the Use of Human Subjects in Research. Each biologically distinct replicate was comprised of one to four donors for UCB and individual donors for BM samples. CD34^+^CD38^−^CD33^−^Rho^lo^c-kit^+^ and CD34^+^CD38^−^CD33^−^Rho^hi^ fractions were selected by sequential Ficoll-Hypaque separation, MACS column depletion, and fluorescence-activated cell sorting as previously described [17]. Postsort analysis demonstrated that sorted populations contained fewer than 1%–2% contaminating cells from the opposing population (see Figure 1).

### Determination of ML–IC frequencies

ML–IC frequencies for UCB samples (*n* = 3) were determined as previously described [17]. An ML–IC was defined as a single cell that gave rise to at least one LTC–IC and one NK–IC. Results are presented as ML–IC frequency ± standard deviation of the mean.

### Processing of RNA samples and oligonucleotide microarray analysis

Total cellular RNA was isolated from UCB (*n* = 5) and BM (*n* = 4) Rho^lo^ and Rho^hi^ cells using the PicoPure RNA Isolation Kit (Arcturus, Mountain View, California, United States) per the manufacturer's instructions. Seven to 10,000 Rho^lo^ and Rho^hi^ cells were sorted directly into 100 μl extraction buffer (XB) provided with the PicoPure RNA Isolation Kit prior to RNA isolation. Labeled cRNA was generated by one round of IVT-based, linear amplification using the RiboAmp OA RNA Amplification Kit (Arcturus) followed by labeling with the Enzo Bioarray HighYield RNA Transcript Labeling Kit (Enzo Life Sciences, Farmingdale, New York, United States) according to manufacturer's instructions. Samples were hybridized to Affymetrix HG-U133 A and B chips (Affymetrix Inc., Santa Clara, California, United States) were washed, and scanned at the University of Minnesota Affymetrix Microarray Core Facility as described in the *Affymetrix GeneChip Expression Analysis Technical Manual.*

### Oligonucleotide microarray data analysis

Affymetrix HG-U133 GeneChips were processed using GeneData Refiner software (GeneData, San Francisco, California, United States) to assess overall quality. Feature intensities for each chip were condensed into a single intensity value per gene using the Affymetrix Statistical Algorithm (MAS 5.0) with τ = 0.015, α1 = 0.04, α2 = 0.06, and a scaling factor of 500. Expression data was analyzed using GeneData's Expressionist and Microsoft Excel (Microsoft, Redmond, Washington, United States). Differential gene expression for comparison of Rho^lo^ versus Rho^hi^ cells was defined by using a paired Student's *t*-test with a threshold of *p*< 0.05, and a paired fold change was used to rank gene lists. Differentially expressed genes were classified according to their respective gene pathways and gene ontologies when available by using the Web-based Affymetrix NetAffx analysis tool (http://www.affymetrix.com) and the National Institutes of Allergy and Infectious Disease Database for Annotation, Visualization and Integrated Discovery analysis tool (DAVID) (http://apps1.niaid.nih.gov/david).

### Microarray Q-RT-PCR confirmation

Labeled cRNA was reverse transcribed to generate cDNA using SuperScript II Reverse Transcriptase (Invitrogen, Carlsbad, California, United States) according to manufacturer's instructions. Q-RT-PCR was performed using the ABI Prism 7000 Sequence Detection System (Applied Biosystems, Foster City, California, United States). Briefly, 3 ng of cDNA was amplified by 40 cycles of a two-step PCR reaction (95 °C for 15 s denaturation and 60 °C for 1 min. annealing/elongation) containing 100 nM gene-specific primers (Table S8) and the SYBR Green PCR Master Mix (Applied Biosystems). Gene expression was normalized using human *ACTB* (also *β-actin*) expression levels. Results are presented as percent expression relative to uninjected clutchmate control embryos ± standard deviation of the mean.

### High-throughput loss-of-function genetic screen in zebrafish

The likely zebrafish orthologs of differentially expressed human genes were identified using Ensembl's gene homology prediction program (http://www.ensembl.org, build Zv3) in combination with comparison of the human protein sequence to the Institute for Genomic Research zebrafish EST database (http://www.tigr.org, release 13–15). The criteria for a likely ortholog was ≥ 40% amino acid identity over the entire length of the protein or ≥ 50% if the fish protein sequence was only partial. MO (Gene Tools, Philomath, Oregon, United States) sequences were designed complimentary to the region of translational initiation of the zebrafish orthologs in order to inhibit protein translation (Table S6) and injected into one- to two-cell zebrafish embryos as previously described [36]. MOs were injected at 1.5, 3, 4.5, and 6 ng into 50–70 embryos derived from mating Tg zebrafish containing a fluorescent DsRed reporter cassette driven by the *gata1* promoter (*gata1:*DsRed Tg) or a fluorescent EGFP reporter cassette driven by the *fli1* promoter (*fli1:*EGFP Tg). MO-targeted (morphant) zebrafish were evaluated for defects in hematopoietic and vascular development compared to uninjected controls from the same embryo clutch at 30 and 48 hpf, using fluorescence microscopy for visualization of DsRed^+^ blood cells and EGFP^+^ vasculature.

### Whole zebrafish Q-RT-PCR confirmation

Total RNA was isolated from five MO-injected zebrafish embryos at 48 hpf with hematopoietic defects, determined based on *gata1:*DsRed^+^ hematopoietic cell production, or five uninjected clutchmate controls using the RNeasy Mini Kit (Qiagen, Valencia, California, United States) according to the manufacturer's instructions. Total RNA was incubated with DNaseI (Invitrogen) to digest contaminating genomic DNA, and reverse transcribed to generate cDNA using SuperScript II Reverse Transcriptase (Invitrogen) according to the manufacturer's protocol. Q-RT-PCR was performed using the ABI Prism 7000 Sequence Detection System (Applied Biosystems). Briefly, 1/20 of the total cDNA from five zebrafish embryos was amplified by 40 cycles of a two-step PCR reaction (95 °C for 15 s denaturation and 60 °C for 1 min. annealing/elongation) containing 100 nM gene-specific primers (Table S8) and the SYBR Green PCR Master Mix (Applied Biosystems). Gene expression was normalized using a zebrafish *gapd* expression level. Results are presented as percentage expression relative to uninjected clutchmate control embryos ± standard deviation of the mean.

### Whole-mount in situ hybridization

The *scl, myod, flk1*, *gata1*, and *cmyb* riboprobes were generated and whole-mount in situ hybridization of zebrafish embryos was conducted as previously described [41].

### Overexpression of human and zebrafish *Sprouty* gene family members

The XhoI and KpnI fragment of the *SPRY1* open reading frame (ORF) (Open Biosystems, Huntsville, Alabama, United States) was cloned into pENTR1A (Invitrogen) and subsequently transferred into a modified pFRM2.1 zebrafish expression vector using the Gateway cloning system (Invitrogen) to create the pFRM2.1–SPRY1 vector. pFRM2.1–SPRY1 was coinjected with pFRM2.1–EGFP at a 5:1 ratio into the yolk/cell interface of one-cell *gata1:*DsRed Tg zebrafish embryos as described for MO injections. Defects in hematopoietic development of EGFP^+^ embryos were analyzed by comparison to embryos from the same clutch injected with pFRM2.1–EGFP alone, using fluorescence microscopy to visualize DsRed^+^ blood cells.

## Supporting Information

Figure S1 Frequency of ML–IC in UCB Rho^lo^ and Rho^hi^ Cell Populations ML–ICs are highly enriched in Rho^lo^ (white bar) compared to Rho^hi^ (black bar) cells from UCB (*n* = 3, *p < 0.05).
(3.6 MB TIF).

Figure S2 Confirmation of Differential Gene Expression Using Q-RT-PCR The Rho^lo^ to Rho^hi^ fold change for *DLK1*, *ABCB1*, *BMP6*, *HELLS*, *CDC25A*, *MAFB*, and *S100A8* was determined using Affymetrix GeneChip analysis (gray bars) and Q-RT-PCR (black bars) to confirm the fidelity of the microarray results for (A) adult BM (*n* ≥ 2) and (B) UCB (*n* ≥ 3).
(7.2 MB TIF).

Figure S3 Gene Ontology Classifications of Conserved Differentially Expressed Genes Percentages of Gene Ontology classifications of the genes differentially expressed between Rho^lo^ and Rho^hi^ cells from both UCB and adult BM (*p* < 0.05 and fold change > 1.5 in either UCB or BM).
(2.7 MB TIF).

Table S1 Probe Sets Differentially Expressed between UCB-Derived Rho^lo^ and Rho^hi^ Cells (*p* < 0.05) (437 KB XLS).

Table S2 Probe Sets Differentially Expressed between Adult BM-Derived Rho^lo^ and Rho^hi^ Cells (*p* < 0.05) (738 KB XLS).

Table S3 Conserved Probe Sets Differentially Expressed between Rho^lo^ and Rho^hi^ Cells from Both UCB and Adult BM (*p* < 0.05 and FC > 1.5 in Either UCB or BM) (75 KB XLS).

Table S4 Fold Changes, *p*-Values, and Average Expression Levels for Genes with Well-Established Roles in HSC Proliferation and Differentiation (17 KB XLS).

Table S5 Comparison of Conserved Rho^lo^-Enriched Genes with Ramalho-Santos et al. [13] Dataset (22 KB XLS).

Table S6 Morpholino Sequences for Targeted Genes (20 KB XLS).

Table S7 Complete Phenotypic Descriptions for the 14 Zebrafish Morphants with Confirmed Blood Defects (19 KB XLS).

Table S8 Q-RT-PCR Primer Sequences (16 KB XLS).

### Accession Numbers

The National Center for Biotechnology Information (http://www.ncbi.nlm.nih.gov/) Entrez Gene accession numbers for the following genes are: ABCB1 (5243), ARMCX2 (9823), BMP4 (652), C12orf2 (11228), CCR7 (1236), Ccr7 (12775), CDKN1A (1026), chd (30161), cmyb (30519), CRYGD (1421), EVI1 (2122), EZH2 (2146), FLJ14917 (84947), flk1 (also known as kdr) (58106), FOXM1 (2305), gata1 (30481), GATA2 (2624), gata2 (30480), hbae1 (30597), HDHD2 (84064), HELLS (3070), HLF (3131), HMGA2 (8091), Hoxb4 (15412), HOXB4 (3214), HSPC039 (51124), IRAK3 (11213), Irak3 (73914), KIAA1102 (22998), KLF5 (688), lcp1 (30583), LEF1 (51176), LMO2 (4005), lmo2 (30332), MAFB (9935), MGC15875 (85007), MRPS6 (64968), myod (30513), NOTCH2 (4853), PIM1 (5292), PRKCH (5583), Prkch (18755), RBPMS (11030), scl (also known as tal1) (30766), SHH (6469), SLC40A1 (30061), SLCO3A1 (28232), SNX5 (27131), SPARC (6678), Sparc (20692), spry4 (114437), SPRY1 (10252), SSBP2 (23635), SUZ12 (23512), ZFHX1B (9839), ZNF165 (7718), and ZNF331 (55422).

The microarray data in this manuscript have been deposited in the GEO database (http://www.ncbi.nlm.nih.gov/geo/, and have been assigned the accession number GSE2666.

## Abbreviations

BM: bone marrow
EGFP: enhanced green fluorescent protein
FGF: fibroblast growth factor
HPC: hematopoietic progenitor cell
hpf: hours postfertilization
HSC: hematopoietic stem cell
ICM: intermediate cell mass
MHC: major histocompatibility complex
ML–IC: myeloid–lymphoid initiating cell
MO: morpholino antisense oligonucleotide
Q-RT-PCR: quantitative RT-PCR
Rho: rhodamine
SCID: severe combined immunodeficiency
TF: transcription factor
Tg: transgenic
UCB: umbilical cord blood
